# Relationship between diabetes and grayscale fractal dimensions of retinal vasculature in the Indian population

**DOI:** 10.1186/1471-2415-14-152

**Published:** 2014-12-01

**Authors:** Behzad Aliahmad, Dinesh Kant Kumar, Marc George Sarossy, Rajeev Jain

**Affiliations:** School of Electrical and Computer Engineering, RMIT University, Melbourne, Australia; Save Sight Centre, Delhi, India

## Abstract

**Background:**

Diabetes mellitus is rapidly increasing in the Indian population. The purpose of this study was to identify changes in the retinal vasculature of diabetic people, ahead of visual impairments. Grayscale Fractal Dimension (FD) analysis of retinal images was performed on people with type 2 diabetes from an Indian population.

**Methods:**

A cross-sectional study comprising 189 Optic Disc (OD) centred retinal images of healthy and diabetic individuals aged 14 to 73 years was conducted. Grayscale Box Counting FD of these retinal photographs was measured without manual supervision. Statistical analysis was conducted to determine the difference in the FD between diabetic and healthy (non-diabetic) people.

**Results:**

The results show that grayscale FD values for diabetic cases are higher compared to controls, irrespective of the gender. It was also observed that FD was higher for male compared with females.

**Conclusions:**

There is difference in the grayscale fractal dimension of retinal vasculature of diabetic patients and healthy subjects, even when there is no reported retinopathy.

## Background

There has been significant increase in the number of diabetic people in the world [1], especially in emerging economies such as India, which has had a very steep rise in diabetes in the younger cohort [2]. The prevalence is significantly higher in some ethnic groups, and often in countries with lower availability of quality healthcare facilities. Untreated diabetes leads to well known complications, such as diabetic retinopathy (DR) [3], and diabetic neuropathy [4]. Diabetic patients are more likely to suffer blindness, neuropathy, ventricular arrhythmia, silent ischaemia, sudden cardiac death and stroke compared with other people.

There have been successes with population screening for diabetes risk assessment [5, 6]. However, opportunistic evaluation accounts for the largest detection of diabetic patients in the low risk population and is largely based on the visit of the person to their primary health provider for other health factors. Work, school or social environments afford another opportunity for such screening. With the reduction in the average age of onset of diabetic patients [7, 8]- now commonly appearing among teenagers- a number of patients go undiagnosed for a significant period [9] and are often diagnosed subsequent to the manifestation of secondary symptoms.

There are a number of screening methods for detecting diabetes. However a large number of people around the globe do not get timely diagnosis and often end up developing DR or other complications. In countries such as Australia and Brazil, one out of every two cases of Diabetes remain undiagnosed [9] until the manifestation of the associated complications. Therefore, there is a need for techniques outside of the clinic that can assess the risk of diabetes in patients in the early stage. This is urgently required in India due to its size and large percentage of diabetes, with more than 62 million individuals currently diagnosed with the disease [10].

Eye fundus imaging allows for non-invasive and in vivo viewing of the retinal vasculature and the microcirculation [11]. With the advances in retinal image analysis, early assessment of the risks associated with a disease condition has become possible. Studies have shown some associations between retinal vascular calibre and Diabetes Mellitus (DM) [12–18], thereby providing an opportunistic modality for diabetes risk assessment. The population based studies have reported association of venules dilation and wider mean retinal arteriolar caliber with diabetes [12, 19]. Another study revealed retinal arteriolar narrowing associated with an increased risk of diabetes in middle-aged persons [20]. Retinal vascular diameter can be considered as a biomarker for diabetes micro-vascular complications [21] and in order to minimise inter-graders variation, the summary statistic of the vessel calibre such as Central Retinal Artery Equivalent (CRAE) and Central Retinal Vein Equivalent (CRVE) has often been used, centred around the Optic Disk (OD) region [22]. Changes to the vasculature shapes, arteriolar branching angle and increased tortuosity have also been reported as indicator of longer duration of diabetes and higher Glycated Haemoglobin (HbA1c) respectively [16].

Population studies have shown that Fractal Dimension (FD) of an eye fundus image shows differences on average between healthy controls and number of disease conditions such as diabetic retinopathy [11, 17] reporting increased retinal vascular FD with increasing odds of diabetic retinopathy [17, 23]. In type 1 diabetes, patients with lower FD are more likely to have proliferative retinopathy [24]. However, no association between retinal FD and any retinal circulatory parameters of the retinal arterioles in patients with type 2 diabetes mellitus has been observed [25].

Current methods to measure FD are based on the binary box-counting (BC) approach which requires image segmentation (binarization), skeletonization and manual correction of the image artifacts which are frequently misidentified as vessels during the segmentation process. This may lead to confounding and biased results due to loss of information during the binarization process [18] and the need for manual supervision which makes it unsuitable for fully-automatic assessments.

In order to overcome the previously mentioned methodological limitations, this study has suggested grayscale based FD analysis of retinal images which can be used for automated analysis, and has shown its association with diabetes. Thus, this technique may be considered for diabetes disease risk assessment at an early stage.

## Methods

### Materials

Experiments were conducted in Department of the Retina, Save Sight Centre hospital located in Delhi. Approval for this study was granted by the Human Research Ethics Committee (HREC) of the Royal Melbourne Institute of Technology (RMIT University), Melbourne, Australia and by the institutional review board at Save Sight Centre hospital in accordance with the declaration of Helsinki (modified 2002). Informed consent was obtained from all participants who had responded to the request advertised in the ‘Save Sight Centre’ in Delhi. For children under 16, the written informed consent was obtained from a parent or legal guardian. It was a prospective nonrandomized study. The participants were classified in two groups; diabetes (case) type 2 and non-diabetes (control). A total of 232 optic disc centred retinal images were taken using Kowa Vx alpha camera (mydriatic and non-mydriatic retinal camera, Kowa, Japan). All photographs were taken in mydriatic mode, with original image resolution being 300dpi (4288 × 2848 pixels) and the camera set to an angle of 30°. These images were cropped into square region of interest (ROI) corresponding to approximately 4 OD diameter cantered at OD centre, and re-sampled to 729 × 485 pixels. A total of 43 images were excluded from the database due to insufficient quality for assessment and FD measurement, leaving 189 (81%) for the analysis.

Participants were defined to be diabetics (cases, n = 23) by their physicians based on fasting and post-prandial glucose levels. The subjects who were not diabetic were considered to be in the ‘control’ group (n = 166). Among all 23 diabetic cases, 5 participants had very mild non-proliferative diabetic retinopathy (NPDR), as identified by the presence of observable damaged blood vessels and micro-aneurism while the retina scan of the remaining 18 did not show any observable retinopathy. The participants belonged to a wide age range of 14 to 73 years old. Diabetic cases were selected based on age (mean ± SD: 52.69 ± 7.90), metric body mass index (BMI (Kg/m2)) (27.60 ± 4.56), blood pressure (mmHg) (systolic: 143.04 ± 19.52, diastolic 81.73 ± 11.83) factors. Participants with history of stroke, hypertension and cardiovascular disorder and other systemic diseases, with or without ocular manifestation, were discarded from this study. Characteristic details of the diabetic and non-diabetic participants have been compared in Table 1.

**Table 1:** **Comparison of characteristics of diabetic cases and control group**

Patients’ characteristics	Diabetic (Case) (n = 23)	Non-Diabetic (control) (n = 166)
	Mean ± SD	Mean ± SD
Age (years)	52.69 ± 7.90	32.50 ± 13.60
Systolic Blood Pressure (mmHg)	143.04 ± 19.52	124.63 ± 15.81
Diastolic blood pressure (mmHg)	81.73 ± 11.83	77.25 ± 14.82
Body Mass Index (Kg/m^2^)	27.60 ± 4.56	24.49 ± 5.32
	**Ratio**	**Ratio**
Gender (Female/Male)	12/11	71/95

### Fractal analysis

FDs of the retinal images were computed using unsupervised Retinal Image and vasculature Assessment Software (RIVAS) v1.0, developed by the authors. After automatically identifying the OD, the software allows examination of different circular regions around the OD at the grayscale level. It provides the examiner multiple analyses options such as measuring vessel caliber summary, vessel calibre of examiner specified segment, tortuosity and FD of the image for different regions. It gives number of different FD options to the examiner; binary and grayscale Box-counting (also known as differential Box-counting (DBC)), Fourier Fractal Dimension (FFD) [26] and Higuchi’s FD [27]. Among these FD techniques, grayscale box-counting does not require image segmentation and is suitable for being performed in an unsupervised manner, and thus is most suitable for automation purpose.

As the first step, the OD of the image is automatically detected using active contour model [28] on the RGB image. A circle is fitted to this boundary and the OD diameter is obtained. A square shaped ROI corresponding to 4 OD diameter is obtained by cropping the image using a binary mask.

Once the ROI is obtained, only the inverted green channel of the eye fundus is used for further analysis as it shows the best vessel to background contrast [29]. As a first step, image enhancement is performed to reduce degrading artifacts and improve the contrast of the vessels from the background performed using the online software (mlvessel v1.3) provided by Soares et al. [26, 29].

The next step is to compute the fractal dimension (FD) of the enhanced grayscale image using DBC. This is a technique developed by Sarkar et al. [30] and is a modification of binary Box-counting with the advantage of being applicable to grayscale images and does not require image segmentation or binarization. In this method, the image is considered in 3D space, where the pixel intensity corresponds to the third dimension (z-axis). The resultant shape is partitioned into grids along the *x*, *y* and *z* axes, where the size of the grid is varied, starting from half the size of the image, and reduced dyadically to a single pixel such that there is a column of cubical boxes covering the grid. The boxes are labelled from 1 till the maximum intensity. The minimum and maximum grayscale intensity (box) of the image corresponding to each grid is identified and the difference between these two is obtained. This is integrated for all the grids at the specific grid ratio, *r* and the number of boxes intersecting image intensity surface corresponding to each *r* (*N*_*r*_), is obtained. The estimate of the fractal dimension is determined from the slope of least square liner fit of log (*N*_*r*_) vs. log (1/*r*). This can be interpreted as the self-similarity within the image, and the value of the FD using DBC method is between 2 and 3, though the typical values of FD computed using DBC are in the range of 2.3 to 2.5, and higher values of FD corresponds to more complex images. The advantage of DBC compared with other FD measures of images is that this does not require the binarization of the images and suitable for automated and unsupervised analysis of the image. It also has the advantage of providing greater separability of FD values at the higher end. For more details, please refer to the work by Sarkar et al. [30].

### Statistical analysis

Statistical analyses were performed using Minitab 16 (Minitab Inc.). The data was first tested for normality by comparison between empirical cumulative distribution function of the data with expected normal distribution (Anderson-Darling, *p* = *0.260*). Multiple ANOVA analyses were performed to test the relationship between retinal vascular FD and two factors; (i) case (diabetes) and control (no diabetes) and (ii) gender factor. The association of FD variations with gender in the presence of diabetes and their corresponding interaction effects was studied using analysis of covariance. To ensure that absolute differences between the case and control were observed so that the technique could be suitable for machine-based analysis.

Multivariate regression analysis was performed to test the association of BMI, age, blood pressure (systolic and diastolic) and gender, as potential confounders, with FD variations. Two different models were constructed to determine the effect of limiting the age factor. In the first model (model 1), FDs corresponding to the entire population (a wide age range of 14 to 73) were modelled by the above predictors plus the diabetes factor. The second model (model 2) was constructed similar to model 1 except the age range which was limited between 50 to 73 years to match the data for this confounding factor. The choice of 50 to 73 was based on prevalence of diabetic cases which was higher among the older age group and to remove skewness from the data due to age factor.

## Results

**Figure 1:**
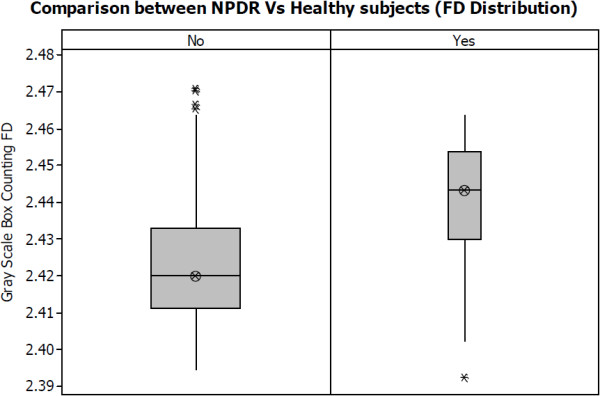
**Box Plot of FD distribution for NPDR and healthy subjects.**

The fractal dimensions (FD) of the retinal vasculature for control and diabetic case are shown in Table 2. The preliminary ANOVA test indicates that the mean FD for diabetic subjects is significantly higher 2.4403 (*p* < 0.001, 95% CI 2.4335-2.4471) compared to the mean FD for the control group (2.4227, *p* < 0.001, 95% CI 2.4201-2.4252). This can also be observed from the box plot of FD values for the two diabetic case and control groups as shown in Figure 1. In this figure, the medians and quartiles are shown separately for case and control groups.

**Table 2:** **Comparison between grayscale BC fractal dimension of control and diabetic cases**

***Diabetes***	***n***	***Box-counting FD (GrayScale)***		
		Mean FD	95% CI	***p*** ^†^
No	166	2.4227	2.4201 - 2.4252	<0.001
Yes	23	2.4403	2.4335 - 2.4471	

^†^α = 0.05.

The FD of the retinal vasculature for all male and all female subjects have been shown in Table 3. The third column shows the Mean while the 95% Confidence Interval (CI) range is in the fourth column. From this table, it is observed that FD for males is significantly higher than the females (*p < 0.001*, males = 2.4375 95% CI 2.4323-2.4427, females = 2.4255, 95% CI 2.4204-2.4306).

**Table 3:** **Comparison between grayscale BC fractal dimensions of males and females**

***Gender***	***n***	***Box-counting FD (GrayScale)***		
		Mean FD	95% CI	***p*** ^†^
Female	83	2.4255	2.4204 - 2.4306	0.001
Male	106	2.4375	2.4323 - 2.4427	

† α = 0.05.

The analysis of interaction between diabetic case and gender has been tabulated in Table 4. The FD of the retinal vasculature for male and female subjects, divided in diabetic case and control have been shown. From this table, it is observed that in general the average FD tends is higher for males compared to the females but no significant interaction between gender and diabetes is observed.

**Table 4:** **Interaction between diabetes and gender**

***Diabetes***	***Gender***	***n***	***Box-counting FD (GrayScale)***		
			Mean FD	95% CI	***p*** ^†^
No	Female	71	2.4203	2.4164 - 2.4242	0.052
	Male	95	2.4251	2.4217 - 2.4284	
Yes	Female	12	2.4308	2.4214 - 2.4402	
	Male	11	2.4499	2.4401 - 2.4598	

^†^α = 0.05.

Table 5 reports the multivariate regression result from the two constructed models. According to model 1 when the entire age range was used, age, gender and diabetes were among the significant predictors of FD variations (all *p* values < 0.05) showing age and gender as possible confounders. In model 2 with increased R-squared (and adjusted R-squared) compared to model 1, the age factor was not found as a significant predictor of the FD (*p* = 0.79), however, diastolic blood pressure was found to be associated with FD change (*p* = 0.003). In both models, FD showed significant association with gender and diabetes factors (all *p* values < 0.05).

**Table 5:** **Multiple regression results for two constructed models**

	Model 1			Model 2		
	14 ≤ age ≤ 73			50 ≤ age ≤ 73		
***Predictor***	***Coef.*** ^*^	***SE*** ^**^	***p*** ^†^	***Coef.*** ^*^	***SE*** ^**^	***p*** ^†^
**Constant**	2.43	0.0101	<0.001	2.35	0.0342	<0.001
**BMI**	−0.0001	0.0002	0.676	0.00016	0.00046	0.722
**Age**	0.0004	0.00009	<0.001	0.00011	0.00041	0.792
**Systolic Blood Pressure**	−0.00012	0.00009	0.232	0.00019	0.00015	0.216
**Diastolic Blood Pressure**	0.00005	0.0001	0.644	0.00070	0.00022	0.003
**Gender**	−0.0075	0.0024	0.003	−0.0126	0.00499	0.016
**Diabetes**	0.0119	0.0042	0.005	0.0100	0.00480	0.034
**R-squared**	20.7%			48.8%		
**R-squared (adjusted)**	18.0%			40.8%		

*Regression Coefficients, **Standard error of coefficients, ^†^α = 0.05.

## Discussion

This research has found that there is a significant difference between the fractal properties of the OD centred eye fundus images of the diabetic subjects with none (n = 18) or mild DR (n = 5) and control subjects, when the FD was computed using unsupervised grayscale box counting method. This suggests that diabetes causes changes in the retinal vasculature complexity before the manifestation of diabetic retinopathy, and vision impairment.

Changes to the retinal vasculature in diabetic patients have been previously reported. The presence of micro-aneurysms and the relationship between the changes in the retinal vasculature calibre and the severity of DR has been recognised by many researchers [19, 31–33]. However, such observations are usually after the manifestation of DR and are not suitable for early stage automated analysis, and recent efforts have been made for machine learning metadata analysis [34]. These are attempts to identify the risk of DR, but none of these studies have tested the relationship between changes to retinal vasculature when there is no visual impairment or signs of DR.

Our research has shown that there is a change in the retinal vasculature prior to the manifestation of DR. This work is significant for two reasons; (i) it shows that there are measurable changes in retinal vasculature ahead of DR, and (ii) that the fractal properties of eye fundus images may be useful for detecting diabetes. Increased grayscale FD of the retinal vasculature has been found to be associated with increased geometric complexity of the retinal vascular branching pattern, reflecting early retinopathy signs in type 2 diabetes which is in agreement with the work by Cheung et al. in which classic binary BC has shown similar association in type 1 diabetes. This work has found the FD variation is significantly associated with diabetes, gender and age factors when studied on a wide age range. However, the result from multiple regression analysis indicated that, in the age limited model (model 2), only diabetes, gender and diastolic blood pressure were strong predictor of FD variations. Comparison between the two constructed models and the result from ANOVA test suggest gender as strong predictor of FD variations with no association with diabetes factor (based on the result from Table 4. This observed strong association of FD variation with age has already been reported using classic binary BC by other studies [35].

One advantage of grayscale FD over the classical binary BC is that it takes the three dimensional pixels intensity information into account. However, in techniques based on binary BC, the intensity information is lost and those corresponding to retinal vasculature are only contrasted from the background pixels. Grayscale FD in contrast, does not require image segmentation [36] and can be directly applied to gray-scale images. Disadvantage of image segmentation is that it may cause loss of vessel caliber information [18] and sometimes requires manual corrections for possible vessel discontinuities as a result of binarization process. Therefore, segmentation can be a potential source of error in FD measurement. Another advantage of grayscale FD over other types of FD calculation is that it does not require manual intervention and can be used for automated analysis of retinal images. Analysis such as automated recognition of diabetes before the manifestation of DR from retinal images may provide a cost-effective method for screening the general population and diagnosing subjects at risk of diabetes at an early age. Retinal photography can be done easily and cheaply as part of a mobile or workplace screening initiative and by a paramedical staff. Such automation methods will make it inexpensive and provide the results immediately. With an increasing number of young people who are suffering from diabetes, such options are essential as many diabetes diagnoses are opportunistic. This technique opens one extra modality which in future it may be very useful for detecting diabetes at an early stage among the younger cohort.

The weakness of this study is that it has been performed on a small sample size and as a result only gender could be considered out of many other possible confounding factors such as age, hypertension, BMI and dyslipidemia. However, for better understanding of the dataset and its limitation, multiple regression analysis was performed to check for possible confounders. Also, the subjects belonged to a wide age range of 14 to 73 years with unbalanced number of control subjects compared to diabetic cases. This wide age range might have led to significant difference between the mean ages of the two population groups and appear as a confounder affecting the analysis. The second regression model in which the age range was narrowed tried to investigate this limitation.

## Conclusions

This research has established that there is a significant difference between the FD of diabetic and healthy people in the Indian population. This supports the generalisation of earlier findings where such a change had been identified for the Australian population [17].

This research has shown that there is association between gray scale FD of retinal vasculature due to diabetes factor, even when the patient has not observed any vision deficiency nor the ophthalmologist observed signs of retinopathy. This outcome suggests that there is a need to conduct longitudinal study to monitor the progress of DR and identify the changes in the fractal properties.
